# Phthalate Exposure and Neurotoxicity in Children: A Systematic Review and Meta-analysis

**DOI:** 10.3389/ijph.2024.1606802

**Published:** 2024-03-25

**Authors:** Evangelia E. Antoniou, Rainer Otter

**Affiliations:** ^1^ MetaAnalyses.com, Hees, Belgium; ^2^ Industrial Petrochemicals Europe, BASF SE, Ludwigshafen, Germany

**Keywords:** behavior, cognition, phthalate, temperament, motor

## Abstract

**Objectives:** This systematic review aims to assess the relationship between prenatal and childhood exposure to phthalates and neurodevelopmental outcomes, identifying periods of heightened susceptibility. Data sources considered studies examining repeated phthalate exposure during pregnancy and childhood on neurodevelopment.

**Methods:** Evaluation included bias risk and study quality criteria. Evidence was synthesized by groups of low and high phthalate molecular weight and exposure measured prenatally and postnatally and outcome measured in childhood. Beta coefficients and their standard errors were extracted, leading to meta-analyses of various neurodevelopmental outcomes: cognition, motor skills, language, behavior, and temperament.

**Results:** Eleven pregnancy and birth cohort studies were identified as relevant. For each phthalate group and outcome combination, there was low or very low evidence of an association, except for prenatal and postnatal phthalate exposure and behavioral development and postnatal exposure and cognition.

**Conclusion:** The estimated effects sizes were relatively small and strong evidence for periods of heightened susceptibility could not be elucidated. No distinction between phthalates of low molecular weight and those of high molecular weight with regards to the outcomes was found.

## Introduction

Phthalates are a group of chemicals that are mainly used as plasticizers in the production of flexible vinyl, which has various uses such as in the production of medical devices, blood bags, toys, wall covers, but also for pharmaceutical coatings [1], food packaging and immediate packaging of medicinal products. It has been hypothesized that levels of phthalates may be associated with health outcomes in children including the risk of allergic reactions such as asthma and eczema, alterations in infant/toddler physical development, and neurodevelopmental defects such as increased internalizing behaviours, decreased child mental and motor development, decreased psychomotor development index (PDI), hyperactivity and poorer social communication in children 0–12 years of age [2–7].

The rationale behind this hypothesis lies in that phthalate exposure occurs in periods of increased vulnerability, such as prenatal, perinatal, and early postnatal life, also known as the brain growth spurt (BGS) period. This period starts during the third trimester of pregnancy up to the first 2 years of life. During this time, the central nervous system goes through different critical developmental processes thus is extremely sensitive to environmental stressors. Phthalates may interfere with one or more developmental processes such as neuronal proliferation, migration, differentiation, synaptogenesis, and myelination, may result in a neurodevelopmental disorder via inducing oxidative stress, DNA damage and apoptosis at the maternal-foetal interface, altering the placenta’s vascularization thus inducing preeclampsia and/or intrauterine growth retardation; increasing prostaglandins therefore weakening the tissue’s integrity; and by interfering with different neuroendocrine systems [8]. These “neuroendocrine disruptors” refer to substances that can interfere with the normal functioning of the endocrine system, particularly affecting the communication between the nervous system and the endocrine system. The hypothalamus-pituitary-gland axis is a crucial component of the endocrine system. Effects on the hypothalamus-pituitary-gland axis may have broad-reaching consequences, as this axis plays a central role in regulating many bodily functions, including growth, metabolism, stress response, and reproductive processes [9].

Di(2-ethylhexyl) phthalate (DEHP) is among the most commonly used phthalates in a worldwide view; other important phthalates include Diethyl- (DEP) and Dimethyl phthalate (DMP) found in fragrances, coatings pills, dyes, and insect repellents; di-n-butyl phthalate (DBP) found in nail polish and cosmetics, varnishes, pills, cellulose acetate plastics; Benzylbutyl phthalate (BBP) found in adhesives, toys and synthetic leather, food containers, and industrial solvents; Diisobutyl- (DiBP) and Di-n-butyl phthalate (DBP) used as plasticizers and in adhesives; and Diisononyl- (DINP) and Diisodecyl phthalate (DIDP) found in plasticized PVC [1, 10]. Sources of phthalates in children or neonates may be breast milk, cow’s milk, infant formulas, plastic food containers, plastic cups or bowls, toys, cups, and even indoor air [1].

In a recent systematic review and meta-analysis Radke et al. reviewed phthalate exposure and its association with cognition, motor skills, behaviour, infant behaviour, and social behaviour; however, contrary to the current hypothesis, only small or indeterminate associations were found, with the exception of motor effects [11]. Moreover, most of the studies included in the review by Radke et al., reported results based on single prenatal urine samples. Nevertheless, there may be periods of heightened susceptibility to phthalate exposures as suggested by one study [12] which investigated the association between neurodevelopmental outcomes and repeated measures of urinary phthalate concentrations during pregnancy and childhood. The topic of this review is to identify potential periods of susceptibility using studies with information on both phthalate exposure during pregnancy and at various time points in childhood and neurodevelopmental outcomes.

## Methods

The PRISMA checklist [13] has been used to guide the information included in the review. The study protocol and data extracted from the included studies can be found online [14] and upon request respectively Table 1.

**TABLE 1:** Inclusion and exclusion criteria (Belgium, 2023).

Category	Inclusion criteria	Exclusion criteria
Population	• Pregnant women and their children	• Population is not human population, e.g., animal studies or *in vitro* or molecular studies
Exposure	• Exposure to one or more of the phthalates during the prenatal period and at the first years of children’s life as determined by	• Phthalate exposure through
	• Urinary metabolite levels of mother and child exposure Types of phthalates include Dimethyl-, Diethyl-, Di-iso-butyl- Di-n-butyl-, Di(2- ethylhexyl)-, Diisononyl-, Di(2-propylheptyl)- or Diisodecyl-	o Ingestion due to mouthing and playing with plastic toys that contain phthalates
		o Environment or house dust (ingestion, dermal contact or inhalation in some circumstances)
		o Due to the use in packaging and food preparation materials
		• Measured concentration in contact medium (e.g., air, dust)
Comparator	• A comparison population exposed to lower levels (or no exposure/exposure below detection levels)	• No comparator
Outcome	• Neurodevelopmental outcomes in children such as (but not limited to) cognition (measure of intelligence, e.g., IQ), learning/memory, motor development, behaviour (internalizing and externalizing, anxiety, ADHD), emotional development, infant behaviour, social behavior and autism spectrum disorder	• Incomplete data on estimates of interest
	• Quantitative data presented in a manner that permitted the calculation of the study summary statistics in a comparable/homogeneous manner across studies	• Data of interest can be found in abstract only without availability of its full text
	• Data collection procedures meet accepted criteria (i.e., sufficient description of study design characteristics) and methodology adequately described	
Study design/Study types	Cohort/follow-up studies	• Cross-sectional, Case-control, Case reports, case series, trials, conference abstracts, opinion, editorials, commentaries, book chapters, protocol, review articles (including literature and systematic reviews)
		• Articles that are not empirical research (i.e., articles that do not have a clearly defined hypothesis or research question that aims to generate new knowledge in this field of research)
Language	English	All Non-English studies
Time frame	Inception to March 2023	
Setting	Global	

### Study Design

The study described herein is a systematic review and meta-analysis of published birth cohort studies.

### Setting

#### Data Sources and Search Strategy

The following databases were searched from inception up to March 2023: PubMed, EMBASE and Web of Science. The concepts to be searched were based on the following PubMed search strategy which was adapted appropriately for use in other databases. Search terms were used in three groups and included: phthalate OR phthalates OR phthalic acid (group 1) AND neurodevelopment, cognition, behavior, learning, emotion, ADHD, anxiety, autism (group 2) AND prenatal, perinatal, neonatal, infant, children, pediatric, maternal, pregnancy (group 3). All MeSH terms were included within the search as well as the free text terms.

Detailed search request has been provided in the Supplementary Tables S1-1 to S1-3. This search was restricted to observational studies conducted only in humans. References from the included papers and previous similar systematic reviews were used to complement adequately the search. Articles from these search and relevant references cited therein were reviewed as eligible for inclusion.

### Data Management

Specific software was used to facilitate and support data management throughout the study. Each program was selected for its specific use in the current systematic literature review (SLR). Literature search results from all three databases stored in Zotero [15], and Covidence software [16] were used for deduplication, screening titles, abstracts and full-texts and recording the exclusion reasons. Included papers were later exported to Zotero and qualitative data were extracted using JotForm. Quantitative data were extracted on excel spreadsheet, prepared, and used for the meta-analysis.

### Selection Process

Studies identified with the structured request were systematically indexed in Covidence software. The first step was to screen titles and abstracts against inclusion/exclusion criteria. As a second step, the eligible articles were then read and assessed for definitive inclusion. Two independent reviewers performed both title/abstract and full-text screening.

### Data Extraction

#### Phthalate Metabolites

Within each study, information was collected at the age that phthalate metabolites exposure was measured (gestational or in (child) years) and the age (in years) that the outcome was measured, potentially leading to multiple exposure-outcome comparisons within a single study. These *exposure windows* can be of cross-sectional nature (when exposure and outcome were measured at the same time) or longitudinal nature (when the outcome was measured at a later time point than the exposure).

Medians (IQR) or means (SD) or geometric means [(95% confidence intervals (CIs)] as reported in each study were extracted (Table 2). Across all studies and types of phthalates, the highest exposure to phthalates was approximately two to three times greater than the lowest exposure level. In addition, information on study design, time of the day the phthalate exposure was measured, gender of the child, and the confounding factors accounted for in the regression analyses were extracted from each study.

**TABLE 2:** Phthalate metabolite concentrations measured in maternal and child urine samples (Belgium, 2023).

Study, year (location)	Phthalate metabolite measured (unit)	Gestational exposure, median (IQR) or mean* (sd) or GM** (95%CI) or GM (GSD)***	Child phthalate exposure; median (IQR) or mean* (sd) or GM** (95%CI)	Age at outcome measure (sample size)/Outcome measure (questionnaire [range])
**Balalian, 2019 (USA)**		**3rd trimester**	**3-years/5-years/7-years**	11-years (n = 209)/Total composite score/Motor skills (BOT-2 [0–88])
	MnBP (ng/mL)	37.1 (18.7–74.5)	48.2 (22–103)/43.7 (18.9–103)/43.5 (21.3–87.4)	
	MBzP (ng/mL)	13.2 (5–26.7)	25.59 (8.5–67.45)/25.6 (8.5–67.4)/22.7 (8.96–61.3)	
	MiBP (ng/mL)	7.6 (4.1–16.4)	14.55 (5.09–30.8)/18.8 (7.5–41)/22.4 (9.8–37.9)	
	MEP (ng/mL)	2.3 (1.2–4.1)	9.2 (3.7–20.5)/11.1 (5.85–21.2)/12.2 (6.35–24.9)	
	MCOP (ng/mL)	131.1 (66.7–302.3)	135.3 (57.55–304.9)/131.3 (61.5–319.4)/100.6 (58.2–231)	
**Factor-Litvak, 2014 (USA)**		**3rd trimester**	**2-years**	7-years (n = 328)/Full scale IQ (WISC-4 [40–160])
	MnBP (ng/mL)	38 (19.4–79.8)	NR^a^	
	MBzP (ng/mL)	14.4 (14.4–30)	NR	
	MEHHP (ng/mL)	21.8 (21.8–47.2)	NR	
	MEHP (ng/mL)	4.9 (4.9–12.4)	NR	
	MEP (ng/mL)	141.5 (141.5–334.1)	NR	
	MiBP (ng/mL)	9.2 (9.2–19)	NR	
**Huang, 2015 (Taiwan)**		**3rd trimester**	**2-3-years/5–6 years/8–9 years/11–12 years**	2–11 years (n = 251); 2–3 years (n = 76); 5–6 years (n = 61); 8–9 years (n = 58); 11–12 years (n = 56)/IQ measured at different time points (BSID-2 [4–21], WISC-3 [40–160], WISC-4 [40–160], WPPSI-R [40–160])
	MMP (ng/mL)	49.84 (40.92–60.71)	14.58 (12.16–17.49)/12.34 (9.73–15.64)/49.84 (40.92–60.71)/8.60 (6.12–12.09)	
	MEP (ng/mL)	66.61 (55.73–79.61)	34.35 (26.78–44.06)/16.18 (12.82–20.43)/13.67 (10.75–17.40)/7.63 (4.99–11.67)	
	MBP (ng/mL)	77.87 (64.84–93.52)	170.12 (145.19–199.33)/111.65 (96.5–129.18)/83.68 (69.70–100.48)/74.86 (65.64–85.37)	
	MBzP (ng/mL)	17.43 (15.15–20.05)	7.45 (5.90–9.42)/14.82 (12.10–18.16)/10.16 (8.00–12.91)/3.21 (2.5–4.10)	
	MEHP (ng/mL)	19.79 (16.38–23.92)	16.26 (13.67–19.35)/13.31 (10.30–17.20)/8.34 (6.28–11.07)/10.07 (8.14–12.44)	
	MEHHP (ng/mL)	8.49 (5.97–12.09)	93.38 (78.78–110.68)/91.30 (72.43–115.08)/42.10 (33.56–52.80)/33.16 (28.98–37.95)	
	MEOHP (ng/mL)	12.97 (9.23–18.21)	65.83 (54.68–79.26)/52.51 (43.14–63.93)/37.07 (29.69–46.28)/24.29 (19.38–30.44)	
Huang, 2019 (Taiwan)**		**3rd trimester**	**2–8 years**	8–14 years (n = 243)/Total internalizing and externalizing behaviour (CBCL Internalizing [0–9]; Externalizing [0–6])
	MMP (μg/g crea)**	52.38 (44.00–62.36) **	NR	
	MEP	63.70 (55.07–73.67) **	NR	
	MBP	67.29 (57.78–78.38) **	NR	
	MBzP	14.99 (13.23–16.98) **	NR	
	MEHP	16.73 (14.46–19.36) **	NR	
**Jankowska, 2019 (Poland)**		**3rd trimester**	**2-years**	7-years (n = 134)/Total difficulties score (SDQ [0–40]); IDS scales [62–135]
	MEP (μg/g crea)	19.4 (10.7–39.5)	9.4 (5.05–21.53)	
	MBzP	0.039 (0.015–0.18)	0.39 (0.16–0.72)	
	MiBP	15.0 (1.7–124.5)	2.3 (0.99–6.31)	
	MEHP	0.19 (0.015–0.53)	0.02 (0.015–0.015)	
	MnBP	4.1 (1.8–12.2)	3.9 (2.45–9.05)	
	MnOP	0.20 (0.015–0.41)	0.02 (0.015–0.23)	
	OH-MEHP	5.1 (0.3–16.7)	2.41 (1.24–5.14)	
	oxo-MEHP	1.6 (0.7–5.0)	1.25 (0.57–2.81)	
	OH-MnBP	5.3 (1.9–10.8)	4.15 (1.88–13.68)	
	OH-MiNP	0.76 (0.5–2.3)	3.07 (0.99–9.55)	
	oxo-MiNP	0.42 (0.015–0.57)	0.20 (0.015–0.56)	
Kim, 2017 (South Korea)***		**2nd trimester**	**6-years**	6-years (175)/Full scale IQ (WISC Korean [55–145]); CPT scales [55–145]
	MBP (μg/g)	30.4 (1.4)	81.8 (1.2)	
	MEHHP (μg/g)	12.5 (1.5)	64.1 (1.3)	
	MEOHP (μg/g)	13.1 (1.4)	47.6 (1.3)	
Ku, 2020 (Taiwan)**		**3rd trimester**	**2-years/5-years/11-years**	2-years (n = 123); 5-years (n = 126); 11-years (n = 122)/Temperament subscales at different ages (CTTS [0–6] and BSQ-C [0–7] and MCTQ-C [0–5])
	MEHP	19.20 (16.69–22.09)	14.77 (−)/11.40 (−)/8.67 (−)	
	MEHHP	8.24 (6.39–10.62)	80.47 (−)/79.80 (−)/30.86 (−)	
	MEOHP	12.41 (9.79–15.73)	58.83 (−)/45.90 (−)/22.70 (−)	
	MBzP	17.09 (15.19–19.22)	6.72 (−)/14.70 (−)/3.12 (−)	
	MBP	75.74 (65.96–86.97)	153.49 (−)/118.73 (−)/51.26 (−)	
	MEP	63.58 (56.17–71.97)	31.65 (−)/19.82 (−)/8.60 (−)	
	MMP	52.34 (44.82–61.13)	15.76 (−)/19.82 (−)/10.98 (−)	
**Li, 2019 (USA)**		**2nd trimester/3rd trimester**	**1-year/2-years/3-years/4-years/5-years/8-years**	5 or 8 years (n = 251)/Full scale IQ (WISC-4 [40–160])
	MnBP (ng/mL)	27.6 (12.6–55.4)/23.2 (8.5–49.2)	−/−/-/22.3 (10.8–42.7)/16.1 (8.9–31.6)/16.2 (8.6–27.7)	
	MiBP (ng/mL)	6.0 (2.2–12.1)/4.1 (1.5–11.0)	−/−/-/11.0 (5.1–22.6)/9.4 (5.5–17.6)/10.4 (5.8–19.7)	
	MBzP (ng/mL)	11.5 (4.3–25.6)/7.8 (3.2–22.8)	10.8 (4.7–26.4)/13.3 (5.4–30.2)/17.2 (5.3–41.5)/12.8 (5.8–28.2)/9.7 (4.6–24.0)/8.4 (4.6–23.8)	
	MCPP (ng/mL)	2.9 (1.4–5.1)/1.7 (0.8–3.5)	4.2 (2.2–9.2)/4.8 (2.2–9.9)/6.3 (3.0–11.8)/4.7 (2.4–11.1)/4.2 (2.1–8.8)/4.1 (2.3–7.8)	
	MEP (ng/mL)	135.3 (54.4–378.8)/114.2 (36.0–318.8)	36.3 (17.3–85.5)/33.4 (14.4–93.7)/40.2 (15.6–82.5)/28.3 (13.1–71.6)/23.0 (12.6–45.9)/22.7 (11.3–53.4)	
	MCOP	−/−	10.1 (4.5–23.5)/11.7 (5.4–24.1)/13.5 (7.2–25.0)/16.6 (7.8–31.5)/23.8 (10.8–47.2)/27.5 (13.8–71.0)	
	MCNP	−/−	4.9 (2.6–9.9)/5.0 (2.3–9.8)/4.6 (2.3–7.5)/4.6 (2.2–8.1)/3.8 (2.5–5.9)/4.6 (2.8–8.2)	
**Li, 2020 (USA)**				
		**2nd trimester/3rd trimester**	**1-year/2-years/3-years/4-years/5-years/8-years**	2–8 years (n = 312)/Internalizing and externalizing problems Behavioral Symptoms Index (BSI [20–80])
	MnBP (ng/mL)	1.87 (1.66–2.20)/1.87 (1.66–2.20)	−/−/-/1.57 (1.48–1.64)/1.42 (1.34–1.48)/1.27 (1.18–1.33)	
	MBzP	1.37 (1.17–1.58)/1.39 (1.22–1.56)	1.66 (1.37–1.75)/1.60 (1.49–1.72)/1.44 (1.33–1.59)/1.30 (1.20–1.48)/1.22 (1.08–1.39)/1.07 (0.92–1.23)	
	MCNP	0.97 (0.70–1.22)/0.97 (0.73–1.19)	1.38 (1.30–1.46)/1.17 (1.10–1.23)/0.99 (0.93–1.05)/0.87 (0.82–0.93)/0.78 (0.72–0.83)/0.76 (0.69–0.83)	
	MCOP	−/−	1.67 (1.62–1.72)/1.58 (1.54–1.62)/1.51 (1.47–1.55)/1.48 (1.43–1.54)/1.47 (1.40–1.54)/1.57 (1.46–1.71)	
	MCPP	−/−	1.35 (1.30–1.41)/1.18 (1.13–1.24)/1.04 (0.99–1.11)/0.92 (0.85–1.01)/0.82 (0.75–0.92)/0.69 (0.56–0.85)	
	MEP	0.38 (0.21–0.58)/0.35 (0.19–0.52)	2.31 (2.12–2.47)/2.05 (1.89–2.21)/1.88 (1.72–2.05)/1.73 (1.56–1.88)/1.63 (1.47–1.79)/1.47 (1.35–1.59)	
	MiBP	2.09 (1.76–2.43)/2.11 (1.82–2.45)	−/−/-/1.28 (1.21–1.35)/1.19 (1.11–1.25)/1.09 (1.04–1.14)	
Polanska, 2014 (Poland)*		**3rd trimester**	**2-years**	2-years (n = 150)/Cognition, language, motor (BSID-3 [50–150])
	MEP (mg/L)	81.3 (274.0)	34.3 (119.5)	
	MiBP	73.8 (141.9)	5.8 (8.8)	
	MnBP	29.4 (140.2)	8.3 (11.9)	
	MBzP	0.2 (0.7)	0.9 (2.3)	
	MEHP	0.4 (0.5)	1.7 (14.7)	
	3OH-MnBP	7.4 (9.7)	15.0 (33.7)	
	5OH-MEHP	15.4 (34.4)	7.2 (17.6)	
	5oxo-MEHP	8.7 (20.2)	4.4 (23.4)	
	oxo-MiNP	0.4 (0.4)	0.5 (1.4)	
	OH-MiNP	4.5 (12.0)	9.3 (20.0)	
	MnOP	0.4 (0.1)	0.9 (7.8)	
	DEHP (μmol/g cre)	0.1 (0.2)	-	
	DiNP (μmol/g cre)	0.02 (0.04)	-	
	DnBP (μmol/g cre)	0.2 (0.4)	-	
**Rolland, 2023 (Norway)**		**2nd trimester/3rd trimester**	**1-year**	2-years (n = 150)/Cognition fixation duration (%); time spent looking at novel face (%); time spent looking at eyes (%); reaction time (ms) (Eyelink software)
	MEP (μg/L)	24.56 (33.29)/19.79 (32.02)	11.35 (14.47)	
	MnBP (μg/L)	10.83 (8.95)/10.88 (9.59)	14.42 (14.19)	
	MiBP (μg/L)	15.05 (11.82)/13.75 (11.26)	11.22 (11.66)	
	MBzP (μg/L)	4.44 (3.86)/3.75 (4.32)	3.46 (5.9)	

^a^ Not reported.

#### Neurodevelopmental Outcome Assessments

Within these exposure windows, beta coefficients (βs) with the corresponding standard error (SE) were extracted or recalculated (based on the reported 95% CIs) for each subscale reported in the studies. Subscales within one test for one neuropsychological category might reflect aspects of another test (i.e., tests are not independent of each other). In our review, we generally followed the recommendations by White et al. [17] on grouping tests into broader categories and thus, our outcome categories included cognition, motor skills, language, behavior, and temperament.

### Data Synthesis and Analysis

For the purpose of the analysis and to summarize the data more efficiently, we divided the phthalates in two groups based on their molecular weight [18] as a) phthalate diesters or phthalate metabolites with low molecular weight (LMW) and b) phthalate metabolites with high molecular weight (HMW). The first group (LMW) consisted of the following phthalate metabolites: 3OH-MnBP (3-OH-mono-n-butyl phthalate), 5OH-MEHP (5-OH-mono(2-ethylhexyl) phthalate), 5oxo-MEHP (5-oxo-mono(2-ethylhexyl) phthalate), DEHP (Di(2-ethylhexyl) phthalate), DBP (Dibutyl phthalate), MBP (Mono-butyl phthalate), MBzP (Mono-benzyl phthalate), MEHHP (Mono-2-ethyl-5-hydroxyhexyl phthalate), MEHP (Mono-2-ethylhexyl phthalate), MEOHP (Mono(2-ethyl-5-oxohexyl) phthalate), MEP (Monoethyl phthalate), MiBP (Monoisobutyl phthalate), MMP (Mono-methyl phthalate), MBP (Mono-butyl phthalate), OH-MEHP (Mono-2-ethyl-5-hydroxyhexyl phthalate), OH-MBP (OH-mono-butyl phthalate), and oxo-MEHP (Mono-2-ethyl-5-oxohexyl phthalate).

The second group (HMW) consisted of the following phthalate diester or phthalate metabolites: DiNP (Di-iso-nonyl phthalate), MCNP (Monocarboxynonyl phthalate), MCOP (Monocarboxyoctyl phthalate), MCPP (Mono(3-carboxypropyl) phthalate), MnOP (Mono-n-octyl phthalate), OH-MiNP (7-OH-mono-methyloctyl phthalate), and oxo-MiNP (7-oxo-mono-methyloctyl phthalate). In some studies, and for reasons explained in each study not all metabolites were used in the regression analyses. As levels of phthalates were reported in all studies to be skewed, log-transformed (either natural, log2-transformed, or log10-transformed) values of phthalates were reported in the studies.

If studies reported beta coefficients (or reported natural log-transformed) for individual subscales within the same neurodevelopmental outcome assessment, these within-study betas were pooled in a fixed-effect meta-analyses using the *metan* command in the statistical software Stata (v.16) in order to obtain one summary beta coefficient result and its corresponding 95% CI. If the studies reported results on summary scales, there was no need to pool results, and these were used as reported in each study. More information on the methodology on the derivation of the summary scales per study is provided in the Supplementary Tables S1-1 to S1-6.

We tabulated the time of phthalate exposure vs. time of outcome measurement separately for LMW and HMW phthalate metabolites and presented the change in the beta coefficient per outcome.

The beta coefficients and standard errors are depicted in Table 4 and in Supplementary Tables S2-5 to S2-8 in Supplementary Material S2. All studies used ln-transformed exposures and betas represent a 1 ln-unit increase, except for Li et al., Li et al., and Kim et al. [12, 19, 20] which used log10-transformation. However, for consistency with the rest of the studies, the betas from these three studies were transformed to represent a 1 ln-unit increase of the exposure per unit increase of the exposure.

In order to facilitate the interpretation of the beta coefficients, we calculated the % change (increase or decrease) based on the range (see Table 2) of the questionnaire scale as reported in each study or on the range of the subscales (see notes under tables and Supplementary Table S1-6), which were used to calculate the pooled beta coefficients. Percentages are shown in Table 4 and in Supplementary Tables S2-5 to S2-8 in Supplementary Material S2 in *italics* in each cell after the beta coefficient and the 95% CI. In the cells where % and estimates are the same, this is indicated with a footnote under the table. We used the following colour coding scheme <=1%; 1-2%; 2-3%; 3-4%; >=5% for the cells to show the percentage change of the outcome per increase of the exposure and to denote a significant association and a positive outcome after phthalate exposure. The following colour coding scheme <=1%; 1-2%; 2-3%; 3-4%; >=5% was used to show the percentage change of the outcome per increase of the exposure and to denote a significant association and a negative outcome after phthalate exposure.

### Study Evaluation

The Newcastle-Ottawa Scale (NOS) [21] for assessing the quality of cohort studies with regards to the selection of the cohort, and exposure and outcome ascertainment was used. Data extraction and quality assessment was performed by two independent reviewers and any discrepancy was resolved by consensus-based discussions among the reviewers to ensure completeness and accuracy (individual study evaluation is provided in the Supplementary Tables S1-1 to S1-4). The Grading of Recommendations Assessment, Development and Evaluation system (GRADE) [22] was used to evaluate the quality of evidence for LMW and HMW phthalate metabolites and the neurodevelopmental outcome (see Supplementary Tables S1-1 to S1-5). The quality rating began with the study design and afterwards was decided whether to downgrade based on five features (risk of bias, unexplained inconsistency, indirectness or lack of applicability, imprecision, and publication bias), or to upgrade based on three features (large magnitude of effect, dose response, consistency across study designs/populations, and consideration of residual confounding). Imprecision was evaluated by *p*-values and CIs, and the number of studies. Indirectness meant discrepancies in populations and measurements of the outcomes among the studies having similar results and evaluation criteria. The confidence ratings were translated into level of confidence in the associations under study according to one of the four statements: 1) High, 2) Moderate, 3) Low or 4) Very Low.

A conclusion of “High confidence” indicates that further research is very unlikely to change confidence in the apparent relationship between exposure to phthalates and the neurodevelopmental outcomes. Contrarywise, a conclusion of “Very Low confidence” suggests that further research is very likely to have an impact on confidence in the association between exposure to phthalates and the outcome.

## Results

### Study Selection

Of the 2,418 studies imported in our database for screening, 1,182 were removed as duplicates.

Of the remaining 1,236 screened studies, 1,120 were considered irrelevant based on title and abstract. Of the remaining 116 articles which were retrieved and assessed in full text, 105 studies were excluded because they did not measure child phthalate exposure measurements (*n* = 74) or were conference abstracts (*n* = 13), or did not report regression coefficients (betas) (*n* = 4), or were duplicate studies (*n* = 6), or animal studies (*n* = 2), or non-English (*n* = 2), or did not report maternal phthalate exposure measurements (*n* = 2) or referred to other exposure (*n* = 2).

**FIGURE 1:**
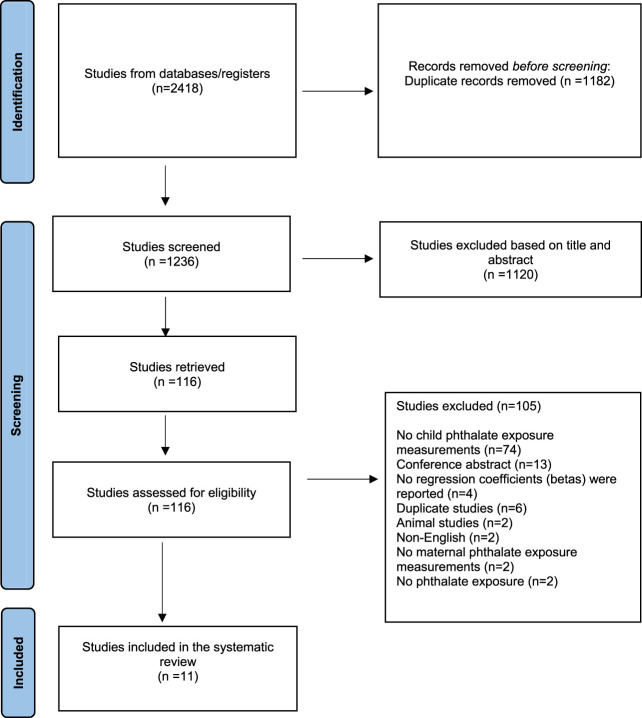
Selection of studies for systematic review (Belgium, 2023).

Finally, 11 pregnancy and birth cohort studies were identified as relevant and included in the systematic review. A flow chart of the study selection can be found in Figure 1.

### Description of the Included Studies

#### Cognition

The relationship between phthalate exposure and cognition was investigated in seven birth cohort studies. These include two studies conducted in the United States [12, 23]; two studies conducted in Poland [24] and three studies, of which one was conducted in Norway [25], one in South Korea [20] and one in Taiwan [26]. All studies included in the review were published between 2014 and 2023. An overview of the phthalate exposures (medians or else reported) measured prenatally and postnatally and the age each outcome was assessed is given in Table 2.

The study by Factor-Litvak et al. [23] was conducted in 328 women, between 18–35 years old, who delivered between 1998 and 2006, and their 7-year old children. These women were enrolled through prenatal clinics associated with Harlem and New York Presbyterian Hospitals and self-identified as either African American or Dominican. Spot urine samples were collected during the third trimester of pregnancy (average 34.0 ± 3.0 weeks, median 33.9) and from children at ages 3 (*n* = 241) and 5 (*n* = 277) years. Samples were analyzed for metabolites of 5 phthalates (DnBP, BBzP, DiBP, DEHP and DEP). The Wechsler Intelligence Scale for Children, fourth edition (WISC-IV) [27] was administered to children at age 7 years.

Information on 251 women from the Health Outcomes and Measured of the Environment (HOME) Study, enrolled from nine prenatal clinics in the Cincinnati, OH, metropolitan area from 2003 to 2006 and their children who were followed-up through age 8 years was used in the study conducted by Li et al. [12]. Maternal urine samples were collected at approximately 16- (*n* = 251) and 26-week (*n* = 245) gestation and from children annually from ages 1–5 years (n ranging from 166 to 212) and at age 8 years (*n* = 218). Urinary concentrations of MEP, MnBP, MiBP, MCPP, MBzP, MCOP, and MCNP were collected. The children’s cognitive abilities were assessed using the Wechsler Preschool and Primary Scale of Intelligence-III (WPPSI-III) [28] and the Wechsler Intelligence Scale for Children, fourth edition (WISC-IV) [27].

A total of 134 mothers and their children from the Polish Mother and Child Cohort (REPRO_PL) recruited between 2007 and 2013 were included in the study by Jankowska et al. [24]. Maternal urine samples were collected from mothers (*n* = 134) during the third trimester of pregnancy (between the 30–34th week) and from children at the age of 2 years (*n* = 123). Prenatal and early childhood exposures were measured in 11 phthalate metabolites: MEP, MiBP, MnBP, OH–MnBP, MBzP, MEHP, 5- OH-MEHP, oxo-MEHP, OH-MiNP, oxo-MiNP, and MnOP. The children’s neurodevelopmental assessment was performed at 7 years of age using the Intelligence and Development Scales (IDS) (*n* = 129) [29].

In the study by Polanska et al. [30] conducted using information from the Polish cohort (REPRO_PL) described above, maternal urine samples from 165 mothers and their children (*n* = 148) were collected during the third trimester of pregnancy (range 30–34 weeks) and postnatally around the 24th month of age (range 23–28 months). The following phthalates were measured: MEP, MiBP, MnBP, 3OH-MnBP, and MBzP, MEHP, 5OH-MEHP, 5oxo-MEHP, OH-MiNP, oxo-MiNP and MnOP. Children’s neurodevelopment was assessed at around the 24th (±1.2) month of age using the Bayley Scales of Infant and Toddler Development, third edition (Bayley-III) [31].

A sub-group of mother-child pairs (*n* = 151) recruited between 2014 and 2017 from the French SEPAGES cohort was included in the study by Rolland et al. [25]. Three spot urine samples (morning, midday, evening) per day over seven consecutive days during the second (median of 18.0 gestational weeks) and third (median of 34.2 gestational weeks) trimesters of pregnancy were collected by the mothers. Postnatally, the mothers collected one urine sample per day over seven consecutive days from their infant around the age of 12 months (median of 12.1 months). Phthalate exposures were measured in the following metabolites: MEP, MnBP, MiBP and MBzP. The neurodevelopment assessment was performed around the child’s 24 months and consisted of an eye tracking experiment.

A total of 175 mother-child pairs enrolled in the on-going cohort named “The Environment and Development of Children” at Seoul National University Hospital between 2008 and 2011, were included in the study by Kim et al. [20]. Maternal phthalate metabolite levels were measured using urine in the second trimester of pregnancy between 14 and 27 weeks (mean of 20 weeks) of gestation and childhood phthalate metabolite levels were measured in the urine of children at age 6 years. The MEHHP, MEOHP, and MBP metabolites were measured. Neurocognitive development was assessed at 6 years using the Korean version of the computerized CPT test Comprehensive Attention Test [32], while children’s IQ was assessed with the Korean Educational Developmental Institute’s Wechsler Intelligence Scale for Children [33].

In the study by Huang et al. [26] a total of 110 mothers, between the ages of 25 and 35, and their children were recruited from the nationwide Taiwan Maternal and Infant Cohort study between 2000 and 2001. Maternal urine samples were collected during the third trimester (range 28–36 weeks) of gestation, while urine samples from children were collected at 2, 5, 8, and 11 years. The MMP, MEP, MBP, MBzP, MEHP, MEHHP and MEOHP metabolites were measured. The assessment of neurocognitive development was performed using the Bayley Scales of Infant Development-II (BSID-II) [31], the Wechsler Intelligence Scale for Children-Version III (WISC-III) [34], the Wechsler Intelligence Scale for Children-Fourth Edition (WISC-IV) [27] and the Chinese version of the Wechsler Preschool and Primary Scale of Intelligence-Revised (WPPSI-R) [35].

#### Behavior and Temperament Traits

Four studies evaluated behavior and temperament traits, three studies [19, 24, 36] on behavior assessment and one study on temperament [37].

In the study by Jankowska et al. [24] (described in previous section) in addition to the assessment of cognitive skills, the children’s behavioral assessment was performed at 7 years of age using the Strengths and Difficulties Questionnaire (SDQ) (*n* = 132) [38].

In the study by Huang et al. [36] 153 mother-child pairs were recruited from the Taiwan Maternal and Infant Cohort Study, between 2000 and 2001. Maternal urine samples were collected during the third trimester of gestation (between the 28th-36th week) and postnatally at the children’s age at 2–3, 5–6 and 8–9 years of age separately. The MMP, MEP, MBP, MBzP, MEHP, MEHHP, MEOHP and ΣMEHP metabolites were measured. The mother completed the Child Behavior Checklist (CBCL) [39] when the children were aged 8–9, 11–12 and 14–15 years.

In the study by Li et al. [19] information on 314 mothers and their children were used from the HOME study. Urinary samples were collected twice during gestation from the mothers and six times from their children at ages 1, 2, 3, 4, 5, and 8 years. Urinary concentrations of MEP, MnBP, MiBP, MCPP, MBzP, MCOP, and MCNP were collected. Children’s behavior was assessed at ages 2, 3, 4, 5, and 8 years using the Behavioral Assessment System for Children-2 [40].

A total of 391 mothers and their children were recruited from the nationwide Taiwan Maternal and Infant Cohort study between 2000 and 2001 in a study conducted by Ku et al [37]. Urine samples from the mothers were collected during the third trimester of gestation (between the 29–40th week) and from the children at 2, 5, 8, and 11 years. Seven phthalate metabolites (MMP, MEP, MBP, MBzP, MEHP, MEHHP, and MEOHP) were measured. Temperament was assessed at ages 2, 5 and 11 years with the Chinese Toddler Temperament Scale (CTTS) [41], the Behavior Style Questionnaire–Chinese version (BSQ-C) [42] and the Middle Childhood Temperament Questionnaire–Chinese version, respectively [43].

#### Language and Motor Skills

In the study by Polanska et al. [30] described in previous section, children’s language and motor skills were assessed with the Bayley Scales of Infant and Toddler Development, third edition (Bayley-III) [31].

In the study by Balalian et al. [44] 209 mother-child pairs were selected from an ongoing birth cohort study (enrolled deliveries between 1999 and 2006) conducted by the Columbia Center for Children’s Environmental Health (CCCEH). Spot urine samples were collected from the mothers during the third trimester (average 33.7 ± 3.2 weeks) and from the children at ages 3, 5 and 7 years. Five phthalate metabolites (MnBP, MBzP, MiBP, MEP and MCOP) were measured. The Bruininks Oseretsky Test of Motor Proficiency short form (BOT-2) [45] was administered at child age 11 to assess gross and fine motor skills.

### Associations Between LMW and HMW Phthalates and Neurodevelopmental Outcomes

In Tables 3-4 and Supplementary Tables S2-5 to S2-8 in Supplementary Material S2 the associations between groups of low and high molecular weight phthalates and neurodevelopmental outcomes are depicted tabulated per exposure timing and time of the outcome measured.

**TABLE 3:** Associations [(betas (95% Confidence Interval)] between low molecular weight phthalates and child cognition (percentages) (Belgium, 2023).

Low molecular weight (LMW)															
Age of child measurement															
Time of exposure measurement	2 years				5–6 years				7 years				8–9 years	11–12 years	2–11 years
2nd trimester	Rolland^a^		Rolland^a^		Kim^b^		Kim^b^		Li, 2019						
	−1.06 (−2.37, 0.24) ^a,c,d^		1.42 (−3.30, 6.14) ^b^		1.14 (−0.13, 2.42) ^a^		0.08 −1.22, 1.38 ^c,d,f,e^		0.04 (−0.39, 0.32) ^a^						
					*(1.3%)*		*(0.1%)*		*(0.04%)*						
3rd trimester	Polanska	Huang, 2015		Rolland^a^	Huang, 2015				Jankowska	Factor-Litvak		Li, 2019	Huang, 2015	Huang, 2015	Huang, 2015
	−0.5 (−0.8, −0.1)	0.45 (−0.36–1.26)		−0.07 (−1.31, 1.18) ^a, c, d^	0.12 (−1.22, 1.46)				**1.45 (0.63, 2.27)** ^**a, b**^	**−0.92 (-1.46, -0.38**) ^a^		0.19 (−0.16, 0.55) ^a^	0.59 (−0.37, 1.55)	−0.25 (−1.29, 0.79)	0.05 (−0.42, 0.53)
	*(0.5%)*	*(0.5%)*		−2.55 (−6.99, 1.89) ^b^	*(0.1%)*				***(1.6%)***	***(-0.8%)***		*(0.2%)*	*(0.5%)*	*(0.2%)*	*(0.04%)*
1-year	Rolland^a^		Rolland^a^						Li, 2019						
	0.37 (−0.75, 1.50) ^a,c,d^		**−7.19 (-11.22, -3.17)** ^**b**^						0.20 (−0.28, 0.68) ^a^						
									*(0.2%)*						
2-years	Polanska		Huang, 2015						Jankowska		Li, 2019				
	−0.4 (−0.9, 0.1)		**−1.12 (-2.03, -0.20)**						−0.02 (−0.12, 0.07) ^a, b^		−0.56 (−1.13, 0.001) ^a^				
	*(0.4%)*		***(-0.9%)***						*(-0.02%)*		*(-0.4%)*				
3-years									Factor-Litvak		Li, 2019				
									**−0.96 (-1.81, -0.11)** ^**a**^		**−0.98 (-1.49, -0.46)** ^a^				
									***(-0.8%)***		***(-1%)***				
4-years									Li, 2019						
									**0.44 (0.06, 0.82)** ^**a**^						
									***(0.3%)***						
5–6 years					Huang, 2015	Kim**		Kim**	Factor-Litvak		Li, 2019				
					0.15 (−0.90, 1.20)	**−3.09 (-5.09, -1.09)** ^**a**^		**−2.73 (-4.76, 0.70)** ^**c,d,e,f**^	0.22 (−0.59, 1.03) ^a^		−0.18 (−0.53, 1.16) ^a^				
					*(0.1%)*	***(-3.4%)***		***(-3.0%)***	*(0.2%)*		*(-0.13%)*				
8–9 years									Li, 2019				Huang, 2015		
									−0.22 (−0.57, 0.12) ^a^				**−3.11 (-4.19, -2.04)**		
									*(-0.2%)*				***(-2.6%)***		
2–11 years															Huang, 2015
															**−1.08 (-1.41, -0.76)**
															***(-0.9%)***
11-years														Huang, 2015	
														−1.12 (−2.30, 0.06)	
														*(-0.9%)*	

Polanska (2014); (total score cognitive, total score language, total score motor) (BSID-III).

Factor-Litvak (2014); a: total scale IQ (WISC-IV).

Huang (2015); total IQ, scores (BSID-II_2years) (WPPSI-R_5 years) (WISC-III_8 years) (WISC-IV_11 years).

Jankowska (2019); a: crystalized intelligence (IDS); b: fluid intelligence.

Kim 2017; a: full scale IQ (WISC); c: omission errors (CPT); d: commission errors (CPT); e: response time (CPT); f: response time variability (CPT).

Li (2019); a: full scale IQ (WISC-IV); cognition was measured at either 5 or 8 years. The results of this study were included in the columns for outcomes at 7 years and 8–9 years of age.

Rolland, 2023; a: fixation duration (%); b: reaction time (ms); c: time spent looking at eyes (%); d: time spent looking at novel face (%) (Eyelink software).

^a^Rolland: Estimates of milliseconds and % are shown in table.

^b^ Kim: exposure transformed to 1 ln-unit.

LMW: 3OH-MnBP, OH-MEHP, 5oxo-MEHP, DEHP, DnBP, MBP, MBzP, MEHHP, MEHP, MEOHP, MEP, MiBP, MMP, MnBP, OH-MEHP, OH-MnBP, oxo-MEHP.

**Bold** estimates indicate that the confidence interval does not contain the null.

Letters after the estimate indicate the test subscales that were pooled together.

Significant associations and positive outcomes <=1%; 1-2%; 2-3%; 3-4%; >=5%

Significant associations and negative outcomes <=1%; 1-2%; 2-3%; 3-4%; >=5%

No associations: grey-shaded.

**TABLE 4:** Associations [(betas (95% Confidence Interval)] between high molecular weight phthalates and child cognition (percentages) (Belgium, 2023).

High molecular weight (HMW)				
Age of child measurement				
Time of exposure measurement	2 years	7 years		8–9 years
2nd trimester		Li, 2019		
		−0.43 (−1.11, 0.24) ^a^		
		*(-0.3%)*		
3rd trimester	Polanska	Jankowska	Li, 2019	
	−0.3 (−1.0, 0.3)	−1.19 (−3.77, 1.39) ^a,b^	0.09 (−0.20, 0.38) ^a^	
	*(-0.3%)*	*(-1.3%)*	*(0.1%)*	
1-year		Li, 2019		
		0.54 (−0.49, 1.28) ^a^		
		*(0.4%)*		
2-years	Polanska	Jankowska	Li, 2019	
	0.1 (−0.5, 0.8)	−0.56 (−3.15, 2.04) ^a, b^	−0.33 (−0.79, 0.14) ^a^	
	*(0.1%)*	*(-0.6%)*	*(-0.3%)*	
3-years		Li, 2019		
		**−0.56 (-1.08, -0.45)** ^a^		
		***(-0.48%)***		
4-years		Li, 2019		
		0.38 (−0.07, 0.83) ^a^		
		*(0.3%)*		
5–6 years		Li, 2019		
		−0.21 (−0.66, 0.24) ^a^		
		*(-0.2%)*		
8–9 years				Li, 2019
				−0.30 (−0.64, 0.05) ^a^
				*(-0.3%)*

Polanska (2014); (total score cognitive, total score language, total score motor) (BSID-III).

Jankowska (2019); a: crystalized intelligence (IDS); b: fluid intelligence.

Li (2019); a: full scale IQ (WISC-IV); cognition was measured at either 5 or 8 years. The results of this study were included in the columns for outcomes at 7 years and 8–9 years of age.

HMW: DiNP, MCNP, MCOP, MCPP, MnOP, OH-MiNP, oxo-MiNP.

**Bold** estimates indicate that the confidence interval does not contain the null.

Letters after the estimate indicate the test subscales that were pooled together.

Significant associations and positive outcomes <=1%; 1-2%; 2-3%; 3-4%; >=5%

Significant associations and negative outcomes <=1%; 1-2%; 2-3%; 3-4%; >=5%

No associations: grey-shaded.

### Cognition and Gestational Phthalate Exposure

From the seven studies (Tables 3, 4) which evaluated phthalate exposure and cognition, two studies [23, 24] showed inverse associations between phthalate exposure during the third trimester and cognition. Specifically, in the study by Factor-Litvak et al. [23] a decrease in cognition measured at 7 years of the child (β = −0.92, 95% CI = −1.46, −0.38) was found for 1 ln-unit increase of the grouped LMW phthalates exposure. In the study by Polanska et al. [30] a decrease in cognition measured at 2 years of the child (β = −0.5, 95% CI = −0.8, −0.1) was found for 1 ln-unit increase of the grouped LMW phthalates exposure.

However, in the study by Jankowska et al. [24] for a 1 ln-unit increase of the LMW phthalate exposure during the third trimester but there was an increase in cognition (β = 1.45, 95% CI = 0.63, 2.27) measured at 7 years. No association was observed for the HMW phthalate exposure and cognition.

In conclusion, small effect sizes were observed for the association between gestational phthalate exposure and cognition and observed mainly with LMW phthalate exposure.

### Cognition and Child Phthalate Exposure

From the seven studies (Tables 3, 4) which evaluated phthalate exposure and cognition, five studies showed inverse associations between child phthalate exposure and cognition.

A decrease in cognition (β = −7.19, 95% CI = −11.22, −3.17) measured at 2 years was observed for 1 ln-unit increase of LMW phthalate exposure measured at 1 year in the study by Rolland et al. [25].

Similarly, in the study by Factor-Litvak et al. [23] a decrease in cognition (β = −0.96, 95% CI = −1.81, −0.11) measured at 7 years was observed per 1 ln-unit increase in LMW phthalate exposure measured at 3 years.

In the study by Li et al. [12] a decrease in cognition (β = −0.98, 95% CI = −1.49, −0.46 and β = −0.56, 95% CI = −1.08, −0.45) measured at 5 or 8 years of age was observed for a 1 ln-unit increase of LMW phthalate exposure and HMW phthalate exposure measured at 3 years respectively. However, an increase in cognition was observed (β = 0.44, 95% CI = 0.06, 0.82) when LMW phthalate exposure was evaluated at 4 years. No association was observed for the HMW phthalate exposure.

In the study by Kim et al. [20] a decrease in cognition (β = −3.09, 95% CI = −5.09, −1.09 and β = −2.73, 95% CI = −4.76, −0.70) measured with two tests at 6 years of age was observed for a 1 ln-unit increase of LMW phthalate exposure measured at 6 years.

Three phthalate exposure times (at 2-years, at 8–9 years, and at 2–11 years) and the corresponding outcome assessment times were evaluated in the study by Huang et al. [26]. A decrease in cognition (β = −1.12, 95% CI = −2.03, −0.20) measured at 2 years was observed for a 1 ln-unit increase of the LMW phthalate exposure measured at 2 years. A decrease in cognition measured at 8-9 and at 2–11 years (β = −3.11, 95% CI = −4.19, −2.04 and β = −1.08, 95% CI = −1.41, −0.76) was observed for 1 ln-unit increase of the LMW phthalate exposure.

In conclusion, large effect sizes were observed for the LMW phthalate exposure group. However, most of the associations were cross-sectional, that is when the exposure and the outcome were measured at the same time points, which limits the interpretation of a long-term effect of the phthalate exposure.

### Behavior and Temperament Traits and Gestational Phthalate Exposure

From the four studies (Supplementary Tables S2-5 to S2-6 in Supplementary Material) which evaluated behavior and temperament traits, two studies on behavior assessment showed some associations with phthalate exposure measured during gestation. No significant change in temperament in relation to gestational exposure was observed in the study conducted by Ku et al. [37].

In the study by Jankowska et al. [24] an increase (β = 0.89, 95% CI = 0.64, 1.13 and β = 0.65, 95% CI = 0.08, 1.22) in behavior problems measured at 7 years of age was observed for a 1 ln-unit increase of the LMW phthalate and HMW phthalate gestational exposure respectively.

In the study by Li et al. [19] an increase (β = 0.48, 95% CI = 0.05, 0.91), of behavior problems measured at some time point between 2 and 8 years was observed for a 1 ln-unit increase of HMW phthalate gestational exposure (second trimester). No association was observed for the LMW phthalate exposure group.

In conclusion, small effect sizes were observed for the association between gestational phthalate exposure and behavior and not consistent between LMW and HMW phthalate exposure groups. Temperament was investigated in one study and very small effect sizes were observed prenatally and postnatally.

### Behavior and Temperament Traits and Child Phthalate Exposure

From the four studies (Supplementary Tables S2-5 to S2-6 in Supplementary Material) which evaluated behavior and temperament traits, three studies on behavior assessment showed some associations with phthalate exposure measured during childhood. No significant associations were observed between phthalate exposure during childhood and temperament.

An increase (β = 0.89, 95% CI = 0.36, 1.42 and β = 1.54, 95% CI = 0.98, 1.03) in behavior problems measured at 7 years was observed in the study by Jankowska et al. [24] for a 1 ln-unit increase of the LMW phthalate and HMW phthalate child exposure measured at 2 years respectively.

Furthermore, in the study by Li et al. [19] an increase (β = 0.46, 95% CI = 0.14, 0.77; β = 1.48, 95% CI = 0.17, 0.79; β = 0.35, 95% CI = 0.03, 0.67) in three (behavioral symptoms index, externalizing and internalizing scales) composite scales measured at some time point between 2 and 8 years was observed for a 1 ln-unit increase of LMW phthalate exposure measured at some point between 1 and 5 years or 8 years of age. For the HMW phthalate exposure group, a 1 ln-unit increase in phthalate exposure measured at some point between 1 and 5 years or 8 years of age was associated with an increase in the behavioral symptoms index and the internalizing problems scale (β = 0.63, 95% CI = 0.34, 0.93; β = 0.14, 95% CI = 0.01, 0.44) respectively.

In the study by Huang et al. [36], however, a decrease in the internalizing problems scale (β = −0.01, 95% CI = −0.02, −0.01) measured at some time point between 8 and 14 years, was observed for a 1ln-unit increase of the LMW phthalate group exposure measured at some time point between 2 and 11 years.

In conclusion, small effect sizes were observed, while more inverse associations were seen for the LMW phthalate exposure group and behavior within longitudinal exposure windows.

### Language and Motor Skills and Gestational Phthalate Exposure

Two studies [30, 44] (Supplementary Tables S2-7, S2-8 in Supplementary Material) evaluated the association between language and motor skills and phthalate exposure during the third trimester. In the study by Polanska et al. [30] a decrease in motor skills measured at 2 years (β = −1.04, 95% CI = −1.44, 0.65) was observed for a 1 ln-unit increase of the LMW phthalate group exposure measured during the third trimester. No significant associations were observed for the HMW phthalate group. No significant associations were observed in the study by Polanska et al. [30] on language development (results not shown).

In the study by Balalian et al. [44] a decrease in motor skills measured at 11 years (β = −0.87, 95% CI = −1.27, −0.47) was observed for a 1ln-unit increase of the LMW phthalate group exposure measured during the third trimester. No significant association was observed for the HMW phthalate exposure group (Supplementary Tables S2–S8 in Supplementary Material S2). In conclusion, very small effect sizes were observed for the association between prenatal exposure and motor skills measured at 2 and 11 years.

### Language and Motor Skills and Child Phthalate Exposure

Two studies [30, 44] (Supplementary Tables S2-7 to S2-8 in Supplementary Material S2) evaluated the association between language and motor skills and phthalate exposure during childhood. No significant associations were observed in the study by Polanska et al. (29) on language development (results not shown).

The study by Balalian et al. [44] showed a decrease in motor skills measured at 11 years (β = −1.66, 95% CI = −3.12, −0.19) for a 1 ln-unit increase of HMW phthalate group exposure measured at 3 years. No association was observed for the LMW phthalate group (Supplementary Table S2-8 in Supplementary Material S2). In conclusion, only the study by Balalian et al. showed an effect in the association between exposure at 3 years and motor skills at 11 years, suggesting (although with a small effect) a longitudinal pattern of the association. Small effect sizes were observed for the association between postnatal exposure and motor skills measured at 2 and 11 years. In addition, no clear pattern on the longitudinal effect of the prenatal exposure can be elucidated.

### Certainty of Evidence

Based on the change in the outcome there is ***moderate certainty*** of confidence in the results which suggest an association between:

• Prenatal and postnatal phthalate exposure and child neurobehavioral development.
• Postnatal phthalate exposure and child cognitive development.

Based on the change in the outcome there is ***low certainty*** of confidence in the results which suggest an association between:

• Prenatal phthalate exposure and motor skills.
• Prenatal phthalate exposure and child cognitive development.

Based on the change in the outcome there is ***very low certainty*** of confidence in the results which suggest an association between:

• Prenatal and postnatal phthalate exposure and child temperament (evidence from one study).
• Prenatal and postnatal phthalate exposure and language development (evidence from one study).
• Postnatal phthalate exposure and motor skills (evidence from one study).

## Discussion

With this systematic review we aimed at evaluating the association between phthalate exposure and child neurodevelopment. To investigate whether there may be periods of heightened susceptibility to phthalate exposure, only studies which assessed phthalate exposure prenatally (maternal exposure) and postnatally (across childhood) were included in this review. The included epidemiological studies examined phthalate exposure individually by analyzing “one chemical at a time.” Nevertheless, in our analysis by classifying phthalates into two exposure groups we investigated a pooled estimate of the effect for each group on the outcome. Consequently, the phthalate exposure measured during gestation and at several time points across childhood and the age of the child when cognition, behavior, temperament, language and motor skills were assessed was tabulated per phthalate group classification.

In general, the data collected and evaluated in the present review do not support a clear pattern of association between prenatal exposure to phthalates and child neurodevelopment. Particularly, from the seven studies which evaluated the association between gestational exposure and cognition, the results of only two studies suggested a significant change in cognition related to LMW phthalate metabolites exposure. In the study by Factor-Litvak et al. [23] there was a decrease in cognition scores while in the study by Jankowska et al. [24] there was even an increase observed in intelligence scores per LMW phthalate exposure. However, in the case of phthalate exposure during childhood the results of five studies [12, 20, 23, 25, 26] showed a decrease in cognitive skills, suggesting that there may be time-dependent associations with postnatal exposure being more strongly associated with phthalate exposure.

In the studies by Rolland et al. [25] and Factor-Litvak et al. [23] exposure of LMW phthalates measured at 1 and at 3 years were associated with decreased cognition measured at 2 and 7 years of age respectively.

In the study by Li et al. [12] where associations of children’s cognitive abilities with urinary phthalate metabolite concentrations were assessed at multiple important periods of neurodevelopment -from the 16th week of pregnancy up to the child’s eighth year of age a decrease in cognitive abilities at age 7 was related to phthalate exposure at age 3. A positive association (i.e., increase in cognitive score at age 7 with increasing phthalate exposure), were observed when the exposure was measured at 4 years, but these were not consistent across the two molecular weight groups and overall, there were not large effects. In addition, in the study by Kim et al. [20] children’s exposure at age 6 was associated with lower cognitive function measured at age 6. Similarly, in the study conducted by Huang et al. [26] children’s postnatal exposure measured at 8 and at some time point between 2 and 11 years of age was inversely related to their behavioral development measured at the same time points as the exposure. Nevertheless, the cross-sectional nature of the exposure and the measurement of the outcomes does not allow us to support the hypothesis that there may be some long-lasting effects related to phthalate exposure.

From the four studies which evaluated the association between phthalate exposure and behavior development, one study showed an increase in behavior problems when LMW phthalate exposure was measured both prenatally and postnatally [24] while another one when exposure was measured only postnatally [19]. No associations were observed in the HMW phthalates. In the study by Huang et al. [36] a positive association was even observed with an increase of LMW phthalate exposure. No association was observed for the HMW phthalates.

Evidence on temperament, which can be considered as the biological precursor of behavioral development, comes from only one study [37] which showed no association between LMW phthalate prenatal and postnatal exposure and temperament measured either during gestation or during childhood.

Moreover, the inconsistent evidence from two studies [30, 44] on motor skills is not sufficient to draw concrete conclusions about the association of prenatal and postnatal exposure and motor skills. Phthalate exposure was associated, in both studies, with a decrease in motor skills only when exposed to LMW during gestation [30, 44], while associations were observed between postnatal HMW phthalate exposure and decreased motor skills in on study [44].

Even though most studies have adjusted for most important confounders, the role of environmental factors such as maternal education, cognitive abilities and the home environment cannot be safely excluded from this association. Furthermore, in our study we constructed summary estimates of the testing instruments used in the original studies. It may be possible that stronger conclusions could be derived by investigating specific subscales with a greater load for the outcome.

In conclusion, the estimated effect sizes were relatively small and strong evidence for periods of heightened susceptibility cannot be elucidated. It’s worth mentioning that a single increment of a 1 ln-unit represents a substantial increase, equivalent to 2.3-fold change in magnitude per unit. This encompassed the entire spectrum of exposure levels from the highest to the lowest, which is almost impossible to encounter in realistic environmental exposures.

Moreover, in general, we found no clear patterns suggesting a dissimilar load of low molecular weight compared to high molecular weight phthalates on the specific outcomes.
