# Supplementary material for: Phthalate Exposure and Neurotoxicity in Children: A Systematic Review and Meta-analysis
Source: Int J Public Health. 2024 Mar 25;69:1606802. doi: 10.3389/ijph.2024.1606802 (PMC10999525; doi:10.3389/ijph.2024.1606802)
Supplement: Supplementary file 1 [file DataSheet2.docx]

# Supplementary material S2

# Phthalate exposure and neurotoxicity in children: A systematic review and meta-analysis

### Table S2-5. Associations [(betas (95% Confidence Interval)] between low molecular weight phthalates and child behavior and temperament (*percentages*) (Belgium, 2023)

| **Low molecular weight (LMW)** | | | | | | | | | | | | |
| --- | --- | --- | --- | --- | --- | --- | --- | --- | --- | --- | --- | --- |
| Age of child measurement | | | | | | | | | | | | |
| Time of exposure  measurement | Behavior  2-8 years | | | | | Behavior  7 years | Behavior  8-14 years | | Temperament  2 years | Temperament  5 years | Temperament  11 years |  |
| 2nd trimester | Li, 2020  0.07 (-0.26, 0.11) ^a^  *(0.1%)*  0.16 (-0.05, 0.36) ^h^  *(0.3%)*  -0.02 (-0.22, 0.17) ^j^  *(-0.04%)* | | | | |  |  | |  |  |  |  |
| 3rd trimester |  | | | | | Jankowska  **0.89 (0.64, 1.13) ^l^ *(0.9%)*** | Huang, 2019  -0.001 (-0.003, 0.002) ^e^  *(-0.02%)*  -0.001 (-0.004, 0.002) ^f^  *(-0.01%)* | | Ku ^a-i^  -0.01 (-0.04, 0.01)  *(-0.2%)* | Ku ^a-i^  -0.07 (-0.15, 0.01)  *(-1.0%)* | Ku ^a-i^  0.004 (-0.07, 0.08)  *(0.1%)* |  |
| 2-years |  | | | | | Jankowska  **1.00 (0.98, 1.03) ^l^ *(1%)*** |  | | Ku ^a-i^  -0.02 (-0.11 0.08)  *(-0.3%)* |  |  |  |
| 5-6 years |  | | | | |  |  | |  | Ku ^a-i^  -0.001 (-0.10, 0.10)  *(-0.01%)* |  |  |
| 2-11 years |  | | | | |  | Huang, 2019  -0.001 (-0.006, 0.003) ^e^  *(-0.25%)* | Huang, 2019  **-0.01 (-0.02, -0.01) ^f^**  ***(-0.1%)*** |  |  |  |  |
| 11-years |  | | | | |  |  | |  |  | Ku ^a-i^  0.02 (-0.02, 0.23)  *(0.4%)* |  |
| 1-5, 8- years* | Li, 2020  **0.46 (0.14, 0.77)** ^a^  ***(0.7%)***  **1.48 (0.17, 0.79**) ^h^  ***(0.8%)***  **0.35 (0.03, 0.67)** ^j^  ***(0.6%)*** | | | | |  |  | |  |  |  |  |
| Huang (2019); e: externalizing problems; f: internalizing problems (CBCL)  Jankowska (2019); l: total difficulties (SDQ)  Li (2020); a: BSI (BASC-2); h: externalising problems (BASC-2); j: internalising problems (BASC-2)  Ku, (2020); a: activity level (BSQ-C @5 years; CTTS @2-years; MCTQ-C @11-years); b: adaptability (BSQ-C @5 years; CTTS @2-years; MCTQ-C @11-years); c: distractibility (BSQ-C @5 years; CTTS @2-years; MCTQ-C @11-years); d: intensity of reaction (BSQ-C @5 years; CTTS @2-years; MCTQ-C @11-years); e:persistence (BSQ-C @5 years; CTTS @2-years; MCTQ-C @11-years); f: positive mood (BSQ-C @5 years; CTTS @2-years; MCTQ-C @11-years); g:rhythmicity (BSQ-C @5 years; CTTS @2-years; MCTQ-C @11-years); h: threshold of responsiveness (BSQ-C @5 years; CTTS @2-years; MCTQ-C @11-years); i: withdrawal (BSQ-C @5 years; CTTS @2-years; MCTQ-C @11-years) (pooled per questionnaire per outcome age)  * measurements were performed either at some point between 1-5 years or at 8 years   \| LMW: 3OH-MnBP, OH-MEHP, 5oxo-MEHP, DEHP, DnBP, MBP, MBzP, MEHHP, MEHP, MEOHP, MEP, MiBP, MMP, MnBP, OH-MEHP, OH-MnBP, oxo-MEHP \| \| --- \| \| **Bold** estimates indicate that the confidence interval does not contain the null \| \| \| Letters after the estimate indicate the test subscales that were pooled together  Significant associations and positive outcomes <=1%; 1-2%; 2-3%; 3-4%; >=5% \| \| --- \|   Significant associations and negative outcomes <=1%; 1-2%; 2-3%; 3-4%; >=5%  No associations grey-shaded \| | | | | | | | | | | | | |
| Table S2-6. Associations [(betas (95% Confidence Interval)] between high molecular weight phthalates and child behavior and temperament (*percentages*) (Belgium, 2023) | | | | | | | | | | | | |
| **High molecular weight (HMW)** | | | | | | | | |  |  |  |  |
|  | Age of child measurement | | | | | | | |  |  |  |  |
| Time of exposure  measurement | Behavior  2-8 years | | | | | Behavior  7 years | Behavior  8-14 years | |  |  |  |  |
| 2nd trimester | Li, 2020  **0.48 (0.04, 0.91**) ^a^  ***(0.8%)*** | | Li, 2020  0.43 (-0.03, 0.89) ^h^  *(0.7%)* | | Li, 2020  0.39 (-0.04, 0.8) ^j^  *(0.6%)* |  |  | |  |  |  |  |
| 3rd trimester |  | | | | | Jankowska  **0.65 (0.08, 1.22) ^l^ *(0.7%)*** | Huang, 2019  0.001 (-0.002, 0.003) ^e^  *(0.02%)* | Huang, 2019  0.001 (-0.002, 0.003) ^f^  *(0.01%)* |  |  |  |  |
| 2-years |  | | | | | Jankowska  **1.54 (0.98, 1.03) ^l^ *(1.7%)*** |  | |  |  |  |  |
| 5-6 years |  | | | | |  |  | |  |  |  |  |
| 2-11 years |  | | | | |  | Huang, 2019  -0.001 (-0.006, 0.003) ^e^  *(0.02%)* | Huang, 2019  -0.002 (-0.01, 0.01) ^f^  *(-0.02%)* |  |  |  |  |
| 11-years |  | | | | |  |  | |  |  |  |  |
| 1-5, 8-years | Li, 2020  **0.63 (0.34, 0.93)** ^a^  ***(1%)*** | Li, 2020  0.13 (-0.19, 0.44) ^h^  *(0.2%)* | | Li, 2020  **0.14 (0.01, 0.44)** ^j^  *(0.2%)* | |  |  | |  |  |  |  |

Huang (2019); e: externalizing problems; f: internalizing problems (CBCL)

Jankowska (2019); l: total difficulties (SDQ)

Li (2020); a: BSI (BASC-2); h: externalising problems (BASC-2); j: internalising problems (BASC-2)

| HMW: DiNP, MCNP, MCOP, MCPP, MnOP, OH-MiNP, oxo-MiNP |
| --- |
| **Bold** estimates indicate that the confidence interval does not contain the null |
| \| Letters after the estimate indicate the test subscales that were pooled together  Significant associations and positive outcomes <=1%; 1-2%; 2-3%; 3-4%; >=5% \| \| --- \|   Significant associations and negative outcomes <=1%; 1-2%; 2-3%; 3-4%; >=5%  No associations: grey-shaded |

### Table S2-7. Associations [(betas (95% Confidence Interval)] between low molecular weight phthalates and child motor skills *(percentages)* (Belgium, 2023)

| **Low molecular weight (LMW)** |  | |
| --- | --- | --- |
| Age of child measurement | | |
| Time of exposure  measurement | 2 years | 11 years |
| 3rd trimester | Polanska  **-1.04 (-1.44, -0.65)** | Balalian  **-0.87 (-1.27, -0.47**)  ***(-0.9%)*** |
| 2-years | Polanska  0.28 (-0.81, 0.25) |  |
| 3-years |  | Balalian  -0.15 (-0.61, 0.31)  *(-0.2%)* |
| 5-6 years |  | Balalian  -0.32 (-0.71, 0.1)  *(-0.4%)* |
| 7-years |  | Balalian  -0.07 (-0.51, 0.37)  *(-0.1%)* |

Balalian (2019); total composite score (BOT-2)

Polanska (2014); total score cognitive, total score language, total score motor (BSID-III). Estimates and percentages shown in the table are the same.

| LMW: 3OH-MnBP, OH-MEHP, 5oxo-MEHP, DEHP, DnBP, MBP, MBzP, MEHHP, MEHP, MEOHP, MEP, MiBP, MMP, MnBP, OH-MEHP, OH-MnBP, oxo-MEHP |
| --- |
| **Bold** estimates indicate that the confidence interval does not contain the null   \| Significant associations and positive outcomes <=1%; 1-2%; 2-3%; 3-4%; >=5% \| \| --- \|   Significant associations and negative outcomes <=1%; 1-2%; 2-3%; 3-4%; >=5%  No associations grey-shaded |

### Table S2-8. Associations [(betas (95% Confidence Interval)] between high molecular weight phthalates and child motor skills *(percentages)* (Belgium, 2023)

| **High molecular weight (HMW)** | | |
| --- | --- | --- |
| Age of child measurement | | |
| Time of exposure  measurement | 2 years | 11 years |
| 3rd trimester | Polanska  -0.56 (-1.28, 0.17) | Balalian  -1.55 (-3.58, 0.48)  *(-1.8%)* |
| 2-years | Polanska  -0.09 (-0.85, 0.68) |  |
| 3-years |  | Balalian  **-1.66 (-3.12, -0.19)**  ***(-1.9%)*** |
| 5-6 years |  | Balalian  -0.96 (-2.56, 0.65)  *(-1.1%)* |
| 7-years |  | Balalian  -0.45 (-1.39, 0.50)  *(-0.5%)* |

Balalian (2019); total composite score (BOT-2)

Polanska (2014); total score cognitive, total score language, total score motor (BSID-III). Estimates and percentages shown in the table are the same

| HMW: DiNP, MCNP, MCOP, MCPP, MnOP, OH-MiNP, oxo-MiNP |
| --- |
| **Bold** estimates indicate that the confidence interval does not contain the null   \| Significant associations and positive outcomes <=1%; 1-2%; 2-3%; 3-4%; >=5% \| \| --- \|   Significant associations and negative outcomes <=1%; 1-2%; 2-3%; 3-4%; >=5%  No associations grey-shaded |
