# Supplementary material for: Phthalate Exposure and Neurotoxicity in Children: A Systematic Review and Meta-analysis
Source: Int J Public Health. 2024 Mar 25;69:1606802. doi: 10.3389/ijph.2024.1606802 (PMC10999525; doi:10.3389/ijph.2024.1606802)
Supplement: Supplementary file 2 [file DataSheet1.docx]

# Supplementary material S1

# Phthalate exposure and neurotoxicity in children: A systematic review and meta-analysis

# Table S1-1 Search strategy in Pubmed (Belgium, 2023)

|  | **Exposure: Phthalate** |
| --- | --- |
| #1 | Phthalic Acids [MeSH Terms] |
| #2 | “phthalic acid” [All fields] |
| #3 | phthalate* [All fields] |

|  | **Outcome: Neurodevelopment** |
| --- | --- |
| #4 | Cognition [MeSH Terms] |
| #5 | Cognitive Dysfunction [MeSH Terms] |
| #6 | Attention [MeSH Terms] |
| #7 | Anxiety [MeSH Terms] |
| #8 | Emotions [MeSH Terms] |
| #9 | Autistic Disorder [MeSH Terms] |
| #10 | "Attention Deficit Disorder with Hyperactivity"[MeSH Terms] |
| #11 | "Psychomotor Performance"[MeSH Terms] |
| #12 | cogniti* |
| #13 | Neuropsych* |
| #14 | "Cognitive impairment" |
| #15 | "Executive function" |
| #16 | "Cognitive ability" |
| #17 | "Motor performance" |
| #18 | "Psychomotor performance" |
| #19 | "cognitive function" |
| #20 | “cognitive performance" |
| #21 | neuro* |
| #22 | learn* |
| #23 | behavio* |
| #24 | emotion* |
| #25 | ADHD |
| #26 | attention |
| #27 | anxiety |
| #28 | autism |

|  | **Population: Pregnant women & children** |
| --- | --- |
| #29 | Pregnancy [MeSH Terms] |
| #30 | Fetus [MeSH Terms] |
| #31 | Gestational Age [MeSH Terms] |
| #32 | Pediatrics [MeSH Terms] |
| #33 | Infant, Newborn [MeSH Terms] |
| #34 | Infant [MeSH Terms] |
| #35 | Child [MeSH Terms] |
| #36 | baby [MeSH Terms] |
| #37 | prenat* |
| #38 | p?ediatr* |
| #39 | fetal* |
| #40 | Fetus |
| #41 | foet* |
| #42 | babies |
| #43 | Baby |
| #44 | infant* |
| #45 | antenat* |
| #46 | neonat* |

# Table S1-2 Search strategy in Embase (Belgium, 2023)

(((((exp "Phthalic Acids"/) OR phthalate* ) OR "phthalic acid" ) OR "phthalic acids" )) AND (((((((((((((((((((((((((exp Cognition/) OR exp "Cognitive Dysfunction"/) OR exp Attention/) OR exp Anxiety/) OR exp Emotions/) OR exp "Autistic Disorder"/) OR exp "Attention Deficit Disorder with Hyperactivity"/) OR exp "Psychomotor Performance"/) OR cogniti* ) OR Neuropsych* ) OR "Cognitive impairment" ) OR "Executive function" ) OR "Cognitive ability" ) OR "Motor performance" ) OR "Psychomotor performance" ) OR "cognitive function" ) OR "cognitive performance" ) OR neuro* ) OR learn* ) OR behavio* ) OR emotion* ) OR ADHD ) OR attention ) OR anxiety ) OR autism )) AND ((((((((((((((((((exp Pregnancy/) OR exp Fetus/) OR exp "Gestational Age"/) OR exp Pediatrics/) OR exp "Infant, Newborn"/) OR exp Infant/) OR exp Child/) OR prenat* ) OR p?ediatr* ) OR fetal* ) OR fetus ) OR foet* ) OR babies ) OR baby ) OR exp baby/) OR infant* ) OR antenat* ) OR neonat* )

# Table S1-3 Search strategy in Web of Science (Belgium, 2023)

(((((("Phthalic Acids") OR phthalate*) OR "phthalic acid") OR "phthalic acids" )) AND (((((((((((((((((((((((((Cognition) OR "Cognitive Dysfunction") OR Attention) OR Anxiety) OR Emotions) OR "Autistic Disorder") OR "Attention Deficit Disorder with Hyperactivity") OR "Psychomotor Performance") OR cogniti*) OR Neuropsych*) OR "Cognitive impairment") OR "Executive function") OR "Cognitive ability") OR "Motor performance") OR "Psychomotor performance" ) OR"cognitive function") OR "cognitive performance") OR neuro*) OR learn*) OR behavio*) OR emotion*) OR ADHD) OR attention) OR anxiety) OR autism )) AND ((((((((((((((((((Pregnancy) OR Fetus) OR "Gestational Age") OR Pediatrics) OR "Infant, Newborn") OR Infant) OR Child) OR prenat*) OR p$ediatr*) OR fetal*) OR fetus) OR foet*) OR babies) OR baby) OR baby) OR infant*) OR antenat*) OR neonat*)

# Table S1-4 Study evaluations using the Newcastle Ottawa Scale for cohort studies (Belgium, 2023)


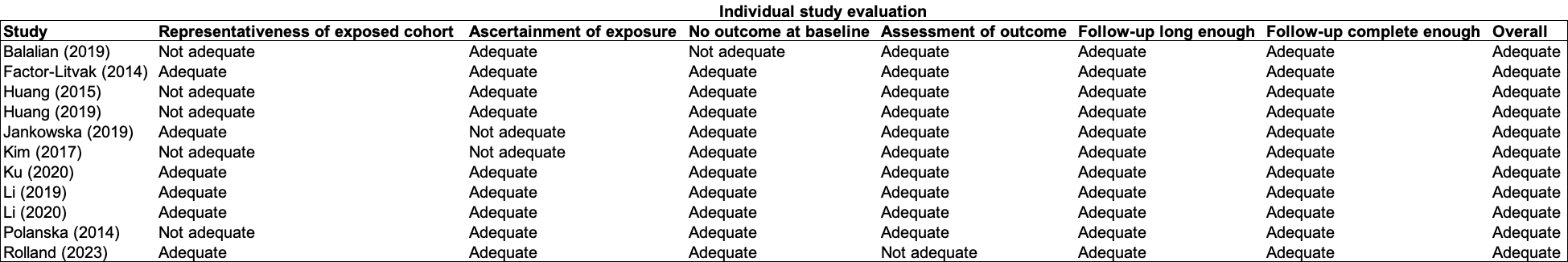


# Table S1-5 GRADE assessment for the association between exposure to phthalates and neurodevelopmental outcomes (Belgium, 2023)


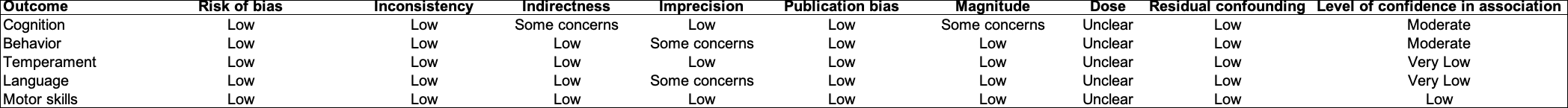


# Table S1-6 Outcomes included in the systematic review and construction of scales for the analysis (Belgium, 2023)

| **Study** | **Outcome measured** | **Questionnaires used**  **(range)** | **Scales used in the analysis as reported in each study** | **Summary scales (estimate) constructed for the analysis** |
| --- | --- | --- | --- | --- |
| Balalian, 2019 | Motor skills | BOT-2 (0-88) | Total composite score |  |
| Factor-Litvak, 2014 | Cognition | WISC-IV (40-160) | Total scale IQ score |  |
| Huang, 2015 | Cognition | BSID-II_2 years (4-21) WPPSI-R_5 years (40-160)  WISC-III_8 years (40-160)  WISC-IV_11 years (40-160) | Total IQ scores per age  BSID-II  WPPSI-R |  |
| Huang, 2019 | Behavior | CBCL  Internalizing (0-9)  Externalizing (0-6) | Internalizing scale^1^/Externalizing scale^2^ |  |
| Jankowska, 2019 | Cognition  Behavior | SDQ (0-40)  IDS* (62-135) | Total difficulties score (SDQ) | Subscales of IDS^3^ were pooled together |
| Kim, 2017 | Cognition | WISC* (55-145)  CPT* (55-145) | Full scale IQ | Subscales of CPT^4^ were pooled together |
| Ku, 2020 | Temperament | CTTS_2 years (0-6)  BSQ-C_5 years (0-7)  MCTQ_11 years (0-5) |  | Subscales^5^ of CTTS, BSQ-C & MCTQ were pooled together per age |
| Li, 2019^ | Cognition | WISC-IV (40-160) | Full scale IQ |  |
| Li, 2020 | Behavior | BASC-2** (20-80) | BSI (BASC-2)/Externalizing problems^6^ (BASC-2)/Internalizing problems^7^ (BASC-2) |  |
| Polanska, 2014 | Cognition/Motor skills/Language | BSID-III (50-150) | Composite scores for cognitive, language, motor development |  |
| Rolland, 2023 | Cognition | Eyelink; eye tracking device (milliseconds & %) | Reaction time (milliseconds) | Subscales^8^ of Eyelink |

BOT-2: Bruininks Oseretsky Test of Motor Proficiency short form

WISC-IV: Wechsler Intelligence Scale for Children, 4th edition

BSID-II: Bayley Scales of Infant Development-2^nd^ edition

WPPSI-R: Wechsler Preschool and Primary Scale of Intelligence-Revised

WISC-III: Wechsler Intelligence Scale for Children-3^rd^ edition

CBCL: Child Behavior Checklist

SDQ: Strengths and Difficulties Questionnaire

CPT: Comprehensive Attention Test

BSI: Behavioral Symptoms Index

BASC-2: Behavioral Assessment System for Children-2

BSID-III: Bayley Scales of Infant Development-3^rd^ edition

^1^ scores on withdrawn, somatic complaints, anxious and depressed were summed (by Huang et al.) to generate the scores of internalizing problems.

^2^ scores on delinquent behavior and aggressive behavior were summed (by Huang et al.) to generate the scores of externalizing problems

^3^ crystalized intelligence, fluid intelligence

^4^ omission errors, commission errors, response time, response time variability

^5^ activity level, adaptability, distractibility, intensity of reaction, persistence, positive mood, rhythmicity, threshold responsiveness, withdrawal

^6^ reflects disruptive behavior problems (i.e., aggression, conduct problems, hyperactivity)

^7^ reflects inwardly directed behavior (i.e., anxiety, depression, somatization)

^8^ fixation duration, time spent looking at eyes, time spent looking at novel face

* range calculated for 3 SD from given mean 100 & SD 15

** range calculated for 3 SD from given mean 50 & SD 10

^increase of the outcome per 1SD reported in the study
